# Multiplex Design of the Metabolic Network for Production of l-Homoserine in Escherichia coli

**DOI:** 10.1128/AEM.01477-20

**Published:** 2020-10-01

**Authors:** Peng Liu, Bo Zhang, Zhen-Hao Yao, Zhi-Qiang Liu, Yu-Guo Zheng

**Affiliations:** a National and Local Joint Engineering Research Center for Biomanufacturing of Chiral Chemicals, Zhejiang University of Technology, Hangzhou, China; b Key Laboratory of Bioorganic Synthesis of Zhejiang Province, College of Biotechnology and Bioengineering, Zhejiang University of Technology, Hangzhou, China; Shanghai Jiao Tong University

**Keywords:** l-homoserine, metabolic engineering, CRISPR interference system, intracellular metabolite profiling, microbial cell factory

## Abstract

l-Homoserine, which is one of the few amino acids that is not produced on a large scale by microbial fermentation, plays a significant role in the synthesis of a series of valuable chemicals. In this study, systematic metabolic engineering was applied to target Escherichia coli W3110 for the production of l-homoserine. Initially, a basic l-homoserine producer was engineered through the strategies of overexpressing *thrA* (encoding homoserine dehydrogenase), removing the degradative and competitive pathways by knocking out *metA* (encoding homoserine *O*-succinyltransferase) and *thrB* (encoding homoserine kinase), reinforcing the transport system, and redirecting the carbon flux by deleting *iclR* (encoding the isocitrate lyase regulator). The resulting strain constructed by these strategies yielded 3.21 g/liter of l-homoserine in batch cultures. Moreover, based on CRISPR-Cas9/dCas9 (nuclease-dead Cas9)-mediated gene repression for 50 genes, the iterative genetic modifications of biosynthesis pathways improved the l-homoserine yield in a stepwise manner. The rational integration of glucose uptake and recovery of l-glutamate increased l-homoserine production to 7.25 g/liter in shake flask cultivation. Furthermore, the intracellular metabolic analysis further provided targets for strain modification by introducing the anaplerotic route afforded by pyruvate carboxylase to oxaloacetate formation, which resulted in accumulating 8.54 g/liter l-homoserine (0.33 g/g glucose, 62.4% of the maximum theoretical yield) in shake flask cultivation. Finally, a rationally designed strain gave 37.57 g/liter l-homoserine under fed-batch fermentation, with a yield of 0.31 g/g glucose.

**IMPORTANCE** In this study, the bottlenecks that sequentially limit l-homoserine biosynthesis were identified and resolved, based on rational and efficient metabolic-engineering strategies, coupled with CRISPR interference (CRISPRi)-based systematic analysis. The metabolomics data largely expanded our understanding of metabolic effects and revealed relevant targets for further modification to achieve better performance. The systematic analysis strategy, as well as metabolomics analysis, can be used to rationally design cell factories for the production of highly valuable chemicals.

## Abstract (precis)

In this study, the bottlenecks that sequentially limit l-homoserine biosynthesis were identified and resolved, based on rational and efficient metabolic-engineering strategies, coupled with CRISPR interference (CRISPRi)-based systematic analysis. The metabolomics data largely expanded our understanding of metabolic effects and revealed relevant targets for further modification to achieve better performance. The systematic analysis strategy, as well as metabolomics analysis, can be used to rationally design cell factories for the production of highly valuable chemicals.

## INTRODUCTION

**FIG 1:**
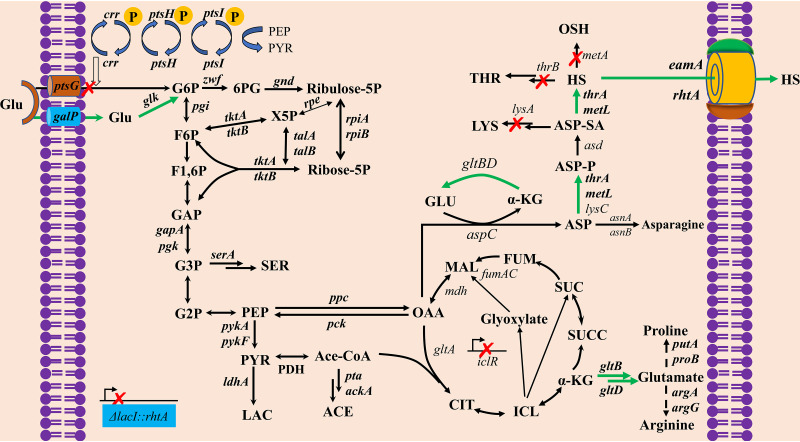
Key metabolic pathway for construction of an inducer-free l-homoserine-producing strain. The red crosses indicate that the genes are disrupted. The green arrows indicate that pathways are overexpressed. Glu, glucose; G6P, glucose 6-phosphate; F6P, fructose 6-phosphate; F1,6P, fructose-1,6-diphosphate; 6PG, 6-phosphogluconolactone; X5P, xylulose 5-phosphate; GAP, glyceraldehyde 3-phosphate; G3P, 3-phosphate-glycerate; G2P, 2-phosphate-glycerate; SER, serine; PEP, phosphoenolpyruvate; Ace-CoA, acetyl-CoA; PYR, pyruvate; LAC, lactate; ACE, acetate; CIT, citrate; ICL, isocitrate; α-KG, α-ketoglutarate; SUCC, succinyl-CoA; SUC, succinate; FUM, fumarate; MAL, l-malate; OAA, oxaloacetate; ASP, l-aspartate; ASP-P, l-aspartate 4-semialdehyde; ASP-SA, l-aspartate 4-semialdehyde; HS, l-homoserine; LYS, l-lysine; THR, l-threonine; OSH, *O*-succinyl homoserine.

A nonessential amino acid for the biosynthesis of l-threonine and l-methionine (1, 2), l-homoserine (l-hydroxybutyric acid), was first synthesized in 1907 by Fischer and Blumenthal. l-Homoserine is also an important precursor for the production of isobutanol, 1,4-butanediol (3), l-phosphinothricin (4), 2,4-dihydroxybutyrate (5), and 1,3-propanediol (6). Therefore, maximizing the productivity of l-homoserine based on new genetic-engineering tools would further extend its potential chemical and biological applications (3, 7). The biosynthesis of l-homoserine has been classified as a linear biosynthetic pathway consisting of glycolysis, the tricarboxylic acid (TCA) cycle, and the aspartate metabolic pathway (Fig. 1). The accumulation of l-homoserine was derived from an l-threonine- or l-methionine-producing strain. It was found that most efforts were focused on regulating gene expression of the l-aspartate metabolic pathway (8, 9). Generally, three aspartokinase isoenzymes (AKI, AKII, and AKIII) that catalyze the first step in l-homoserine biosynthesis were overexpressed to enhance the carbon flux to l-homoserine production. In addition, the transporter system was enhanced to improve survivability under the stress of l-homoserine (10). Due to the lack of optimization upstream of glycolysis, as well as branched pathways (11), l-homoserine was produced with a low yield by mutant and metabolically engineered strains, including Corynebacterium glutamicum (12) and Escherichia coli (13, 14). Until now, the optimization of each gene participating in different pathways and the identification of bottlenecks limiting l-homoserine biosynthesis in E. coli have rarely been reported.

Currently, pathway design and optimization are essential to obtain microbial cell factories with excellent performance for industrial production of commercial chemicals (15, 16). A series of attempts have been made to construct novel producers for biobased chemicals as the technologies in support of synthesizing new materials and manipulating genes have matured (17–19). Indeed, some strategies have been applied to optimize the biosynthetic pathways, which consist of large numbers of genes, or to reprogram gene expression to manipulate complex phenotypes. Prominent examples of such methods include the multidimensional heuristic process (MHP) (20), multiplex navigation of global regulatory networks (MINR) (21), and multibranched and multilevel regulated biosynthetic pathways (MBMRPs) (22), which rapidly engineer organisms with desired capabilities without the trial and error of iterative experimentation. On the other hand, “omics” profiling technologies, such as metabolomics, have facilitated an overview of cell metabolism, allowing a more in-depth insight into intracellular mechanisms in modified-organism analysis (23).

Here, the initial l-homoserine-producing strain was obtained by blocking the degradative and competitive pathways and overexpressing *thrA* (encoding homoserine dehydrogenase) based on an *O*-succinyl homoserine-producing strain (24). Then, rational design, including reinforcement of the transport system, redirection of the carbon flux, the CRISPR interference (CRISPRi) system, and module integration strategy, were used to construct an l-homoserine-producing strain. The iteratively modified strain HS33 could produce 7.25 g/liter l-homoserine from glucose in a shake flask. Metabolomics analysis further revealed the intracellular behaviors (metabolic differences) in response to genetic modifications to direct carbon to l-homoserine formation. Based on the metabolomics analysis, the anaplerotic route afforded by pyruvate carboxylase was introduced to direct carbon toward l-homoserine, which resulted in accumulating 8.54 g/liter l-homoserine (0.33 g/g glucose, 62.4% of the maximum theoretical yield) in shake flask cultivation. Finally, 37.57 g/liter l-homoserine was produced under fed-batch fermentation, with a yield of 0.31 g/g glucose.

## RESULTS

### Construction of an initial l-homoserine-producing strain.

**FIG 2:**
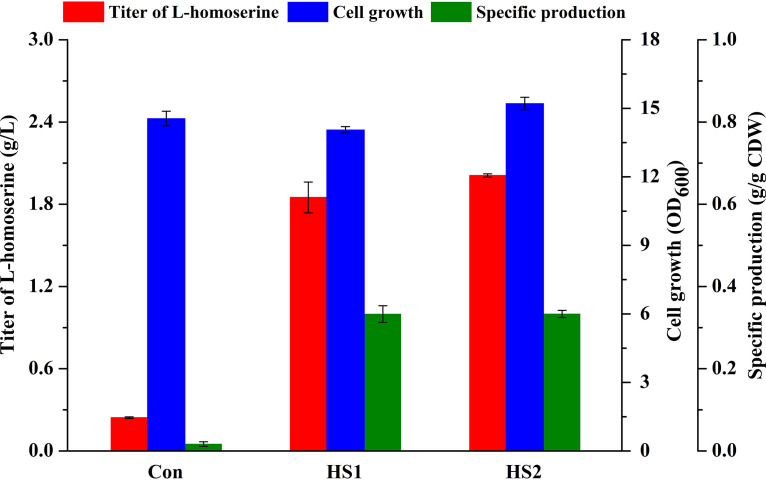
Comparison of fermentation performances of the engineered strains. Cells were cultured in MS medium at 28°C for 48 h. Con, control. The error bars represent SD.

The “pull-push-block” strategy is an efficient method to engineer microorganisms involved in biosynthesizing target products by modifying metabolic networks (25, 26). In our previous study, the E. coli strain ΔJIB* Trc*metL* with overexpression of *metL* and deletion of the *metJ*, *metI*, and *metB* genes was constructed to produce *O*-succinyl homoserine from l-homoserine and succinyl-coenzyme A (CoA) (24). To construct the basic l-homoserine-producing strain HS1, l-homoserine-converting pathway-related genes (*thrB*, encoding homoserine kinase, and *metA*, encoding homoserine *O*-succinyltransferase) were successively deleted to “block” l-homoserine degradation. Then, the l-homoserine-converting pathway was further strengthened by overexpression of *thrA* to “push” the carbon flux to l-homoserine production. Subsequently, the lysine-auxotrophic strain HS2 was generated by deleting *lysA* to investigate the effect of eliminating a precursor competing metabolic pathway on l-homoserine production. As shown in Fig. 2, HS1 could accumulate 1.85 g/liter of l-homoserine in MS (minimal salt) medium. The disruption of l-lysine biosynthesis increased the production of HS2 to 2.01 g/liter with the same specific production of 0.33 g/g cell dry weight (CDW) when l-lysine (0.025 g/liter, optimized amount) was added (see Fig. S1 in the supplemental material).

### Modification of the transport system for improvement of the l-homoserine titer.

**FIG 3:**
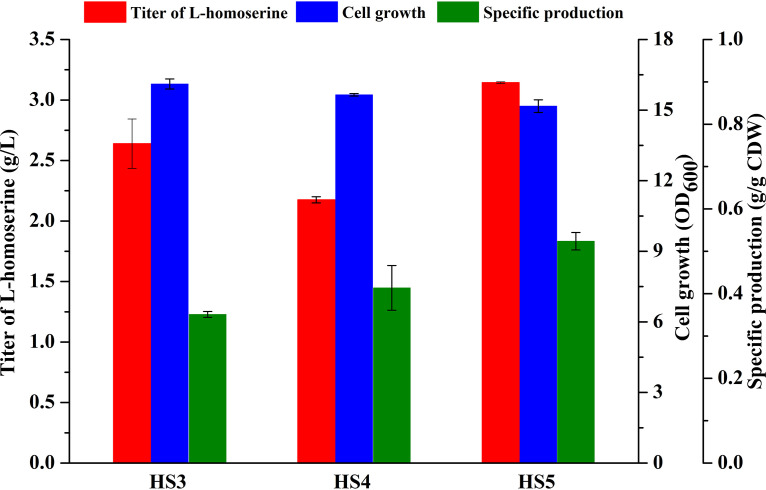
Effect of transport system (RhtA and EamA) modification on l-homoserine production. Cells were cultured in MS medium at 28°C for 48 h. The error bars represent SD.

Based on observations that l-homoserine inhibits the activity of aspartokinase (encoded by *metL*) and l-glutamate dehydrogenase (encoded by *gdhA*) (27, 28), toxicity stress arising from gradual accumulation of product results in repressing cell growth and product yield (29, 30). Therefore, strengthening the capability of the l-homoserine transport system and the transformation of other toxic intermediate metabolites are top priorities. *rhtA*, encoding the inner membrane transporter that is involved in the export of l-homoserine (31), was overexpressed chromosomally by replacing the native promoter with the *trc* promoter to obtain strain HS3 (Trc-*rhtA*). Strain HS3 increased the production of l-homoserine by 30.9% to 2.63 g/liter (Fig. 3). It was reported that overexpression of *eamA* confers resistance to toxic chemicals (32). In order to further increase the l-homoserine export capacity and relieve the growth burden of homoserine-producing strains to enable survival, the native promoter of the *eamA* gene was replaced by the *trc* promoter in strain HS4 (Trc-*eamA*). Batch cultivation of HS4 resulted in the production of 2.17 g/liter l-homoserine. Moreover, two *rhtA* gene copies (the native *rhtA* gene and replacement of the *lacI* gene) and *eamA* were overexpressed under the *trc* promoter in the chromosome to construct strain HS5 (Δ*lacI*::Trc-*rhtA* Trc-*rhtA* Trc-*eamA*). Under batch culture, strain HS5, with modification of the transport system and construction of a constitutive expression system, could produce 3.14 g/liter l-homoserine, which was 54.2% higher than strain HS2 production. In addition, the specific production of strain HS5 was also increased (Fig. 3), which confirmed that enhancing the transport system was beneficial for the improvement of l-homoserine productivity.

### Effect of a glyoxylate shunt on l-homoserine production.

**FIG 4:**
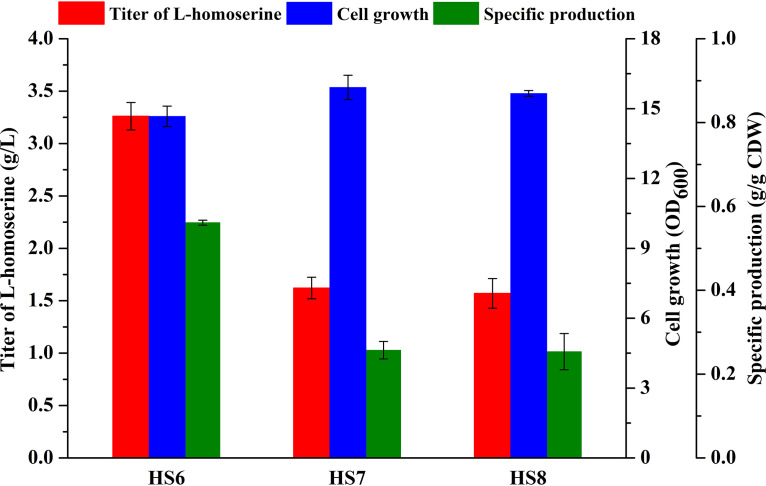
Effects of TCA cycle perturbation on fermentation performances of different engineered strains. Cells were cultured in MS medium at 28°C for 48 h. The error bars represent SD.

The deletion of the *iclR* gene can activate the glyoxylate shunt, which increases the availability of malate as the precursor of oxaloacetate (2, 33). To further direct the carbon flux to l-homoserine, the transcriptional regulator IclR was removed to construct strain HS6. As shown in Fig. 4, the final l-homoserine concentration obtained in strain HS6 with *iclR* deleted was equal to that in the control strain. Additionally, the AspA-catalyzed reaction is an efficient alternative to the AspC-catalyzed reaction in the conversion of fumarate and oxaloacetate to l-aspartate (34, 35). Therefore, the deletion of fumarase genes (*fumA*, *fumC*, and *fumB*) and overexpression of *aspA* were implemented. Under batch culture, both strains HS7 (with *fumAC* deleted) and HS8 (with *aspA* overexpressed) showed lower l-homoserine production than HS6 (Fig. 4).

### Repression of candidate genes by the CRISPRi system to further enhance l-homoserine production.

**FIG 5:**
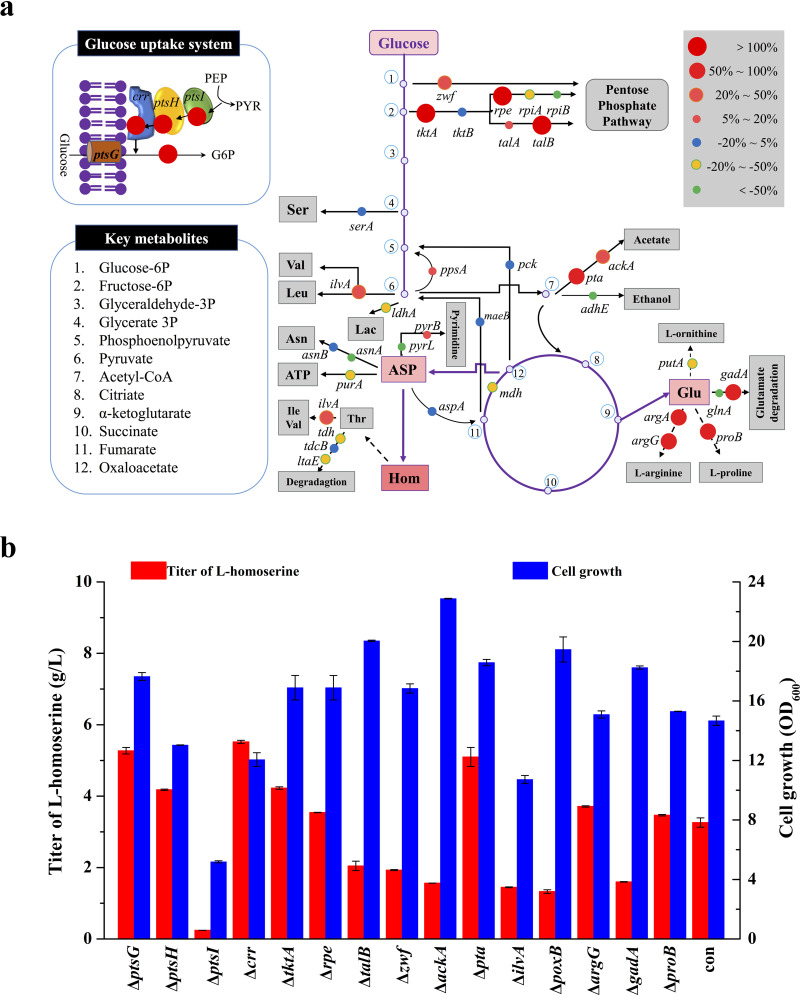
CRISPR-dCas9-based strategy for systematic screening and fine tuning of gene expression for l-homoserine overproduction. (a) Schematic of CRISPR-dCas9-based gene regulation in the l-homoserine biosynthesis pathway. The 39 target genes, including glycolysis, by-products, and branch pathways, were investigated in this study. The results were drawn from combined data for the single gene target among the genes. Relative changes in l-homoserine yield compared with the negative control (HS5 harboring a pTarget-null plasmid without N20 sequence) are represented as colored circles. Blue circles represent no significant change (−20% to 5%) in l-homoserine production compared to the negative control. Targets that increased l-homoserine production are shown in red. (b) Cell growth and homoserine production in deletion of positive targets. Control (con) refers to strain HS6. The cells were cultured in MS medium at 28°C for 48 h. The error bars represent SD.

To identify the target genes to be further manipulated, the CRISPRi system for sequence-specific control of gene expression was used on the l-homoserine production strain HS6. Specifically, the homoserine-forming pathway was partitioned into three modules, which were separated at GAP (d-glyceraldehyde 3-phosphate) and OAA (oxaloacetate). These metabolic nodes are involved in the glycolytic pathway, by-product production, and amino acid biosynthesis. The results for strains with target gene downregulation were measured and compared with the control harboring the pTarget-null plasmid without an N20 sequence [HS6(pdCas9, pTarget-null)]. As shown in Fig. 5a, 39 genes were chosen to study the effects of target gene repression on l-homoserine production. Compared with the control group, HS6(pdCas9, pTarget-null), the strains with single guide RNAs (sgRNAs) targeting genes exhibited differences in the accumulation of l-homoserine. The l-homoserine production of strains with sgRNA-directed downregulation of *ptsH*, *ptsI*, *crr*, *ptsG*, *tktA*, *rpe*, *talB*, *argA*, *argG*, *proB*, and *gadA* in three modules were increased by more than 100%. On the other hand, the sgRNAs targeting *zwf*, *pta*, and *poxB* also increased the accumulation of l-homoserine by 50% to 100% (Fig. 5a).

On the basis of these results, candidate genes with promising positive targets for l-homoserine yields were selected for further individual deletion. As such, *ptsG*, *ptsH*, *ptsI*, *crr*, *tktA*, *rpe*, *talB*, *zwf*, *ackA*, *pta*, *ilvA*, *poxB*, *argG*, *gadA*, and *proB* were removed individually based on strain HS6 to construct a series of novel l-homoserine producers (strains HS9 to HS23). As shown in Fig. 5b, the cell growth of these engineered strains was variable; in particular, growth of the strain with deletion of *ptsI* was seriously inhibited. Additionally, the results showed that the strains with individual deletions of *ptsG*, *ptsH*, *crr*, *tktA*, *rpe*, *pta*, *argG*, and *proB* accumulated higher l-homoserine content than the control strain. In particular, strains HS9 (Δ*ptsG*) and HS12 (Δ*crr*) with perturbation of the phosphotransferase system (PTS) showed l-homoserine production at 5.27 g/liter and 5.52 g/liter, respectively, which was higher than that of other mutations. These positive genes, which were screened out by the CRISPRi system, should be further investigated to identify the rate-limiting steps blocking l-homoserine biosynthesis.

### Restoration of glucose uptake by non-PTS sugar transporter modification.

**FIG 6:**
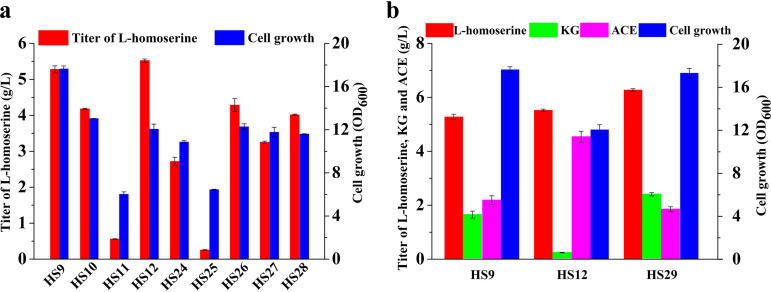
Modification targets for enhancement of the glycolysis pathway. (a) Effects of disrupting the PTS and pentose phosphate pathway on l-homoserine production and cell growth. (b) Effects of modification of a non-PTS glucose uptake system on cell growth and homoserine production. KG, ketoglutarate; ACE, acetate. Cells were cultured in MS medium at 28°C for 48 h. The error bars represent SD.

On the basis of the above-mentioned results, the effects of the iterative genetic modifications of the selected genes not participating in l-homoserine biosynthesis were investigated. In this study, combinational genetic perturbation targeting the PTS and the pentose phosphate pathway was performed. To provide ample upstream pathway strength, a new set of five strains (HS24 to HS28) were engineered on the basis of perturbation of the PTS equilibrium, on which iterative genetic modifications were carried out between the PTS and the pentose phosphate pathway. Unexpectedly, the results showed that the l-homoserine yield was reduced in these further engineered strains (Fig. 6a). Given the differences in cell growth and major by-product accumulation between HS9 and HS12, the Δ*ptsG* strain (HS9) was chosen as the candidate for further investigation (Fig. 6b). Due to the decrease in glucose uptake caused by the *ptsG* gene deletion, the promoter region of the *glk* gene (encoding glucokinase) was replaced with the *trc* promoter, and inactivation of *galR* led to constitutive expression of a non-PTS sugar transporter to restore glucose transport capacity for efficient l-homoserine production (see Fig. S2 in the supplemental material). The l-homoserine titer observed with the HS29 strain was 6.27 g/liter, which was a 19.0% increase compared to the HS9 strain. The major by-products, including acetic acid and α-ketoglutarate, were produced at 1.86 g/liter and 2.39 g/liter, respectively, in flask fermentations.

### Flux reinforcement by enhancing circulation of the amino donor.

**FIG 7:**
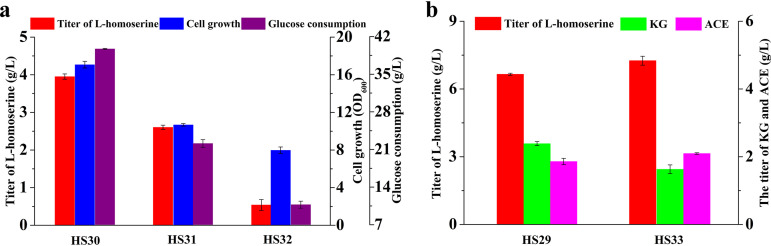
Modification of metabolic flux at the glutamate node. (a) Comparison of l-homoserine production, cell growth, and glucose consumption in different engineered strains. (b) Effects of *gltBD* operon overexpression on l-homoserine and by-product production. The error bars represent SD.

It should be clear that glutamate functions as the amino donor for the transformation of oxaloacetate into l-aspartate. On the other hand, α-ketoglutarate would be accumulated from l-glutamate under conditions of nitrogen limitation. Not only is α-ketoglutarate responsible for catalyzing the first reaction in ammonia assimilation, it coordinates carbon and nitrogen utilization by rapid modulation of the glycolytic flux to alter import of glucose and consumption of phosphoenolpyruvate (PEP) (11). Therefore, in order to convert α-ketoglutarate into l-glutamate, strain HS30 was constructed by replacing the wild-type promoter of the *gltBD* operon (encoding l-glutamate synthase) with a strong *trc* promoter in strain HS6, and the l-homoserine yield was increased by 23.1% to 3.95 g/liter. This showed that the amino donor (l-glutamate) supply was considered another bottleneck in the biosynthesis of l-homoserine. Additionally, another set of two strains (HS31 and HS32) were constructed by deleting *argG* and *proB* based on the *gltBD* gene upregulation strain HS30 to increase the availability of intracellular l-aspartate and l-glutamate, respectively. However, not only were cell growth and glucose consumption severely repressed, but less accumulation of l-homoserine was observed, especially for strain HS32 (Fig. 7a).

These observations prompted the construction of strain HS33, which was iteratively modified by upregulating the expression of l-glutamate synthase based on strain HS29 to remove the deeper rate-limiting step. The successful overexpression of selected genes was subsequently verified by reverse transcription-quantitative PCR (RT-qPCR) (see Fig. S3 in the supplemental material). Notably, the observed l-homoserine production by strain HS33 was increased by 15.6% to 7.25 g/liter without affecting cell growth and the consumption of glucose (Fig. 7b). Also, the concentration of the by-product *α*-ketoglutarate was further decreased to 1.63 g/liter (Fig. 7b), indicating that upregulation of the *gltBD* operon could effectively facilitate the conversion of α-ketoglutarate into l-glutamate. It was concluded that the multidirectional control of metabolic pathways associated with metabolic strategies and CRISPR-based transcriptional regulators enabled efficient carbon utilization and recovery of the amino donor for production of l-homoserine.

### Metabolic variation response to iterative genetic modifications.

**FIG 8:**
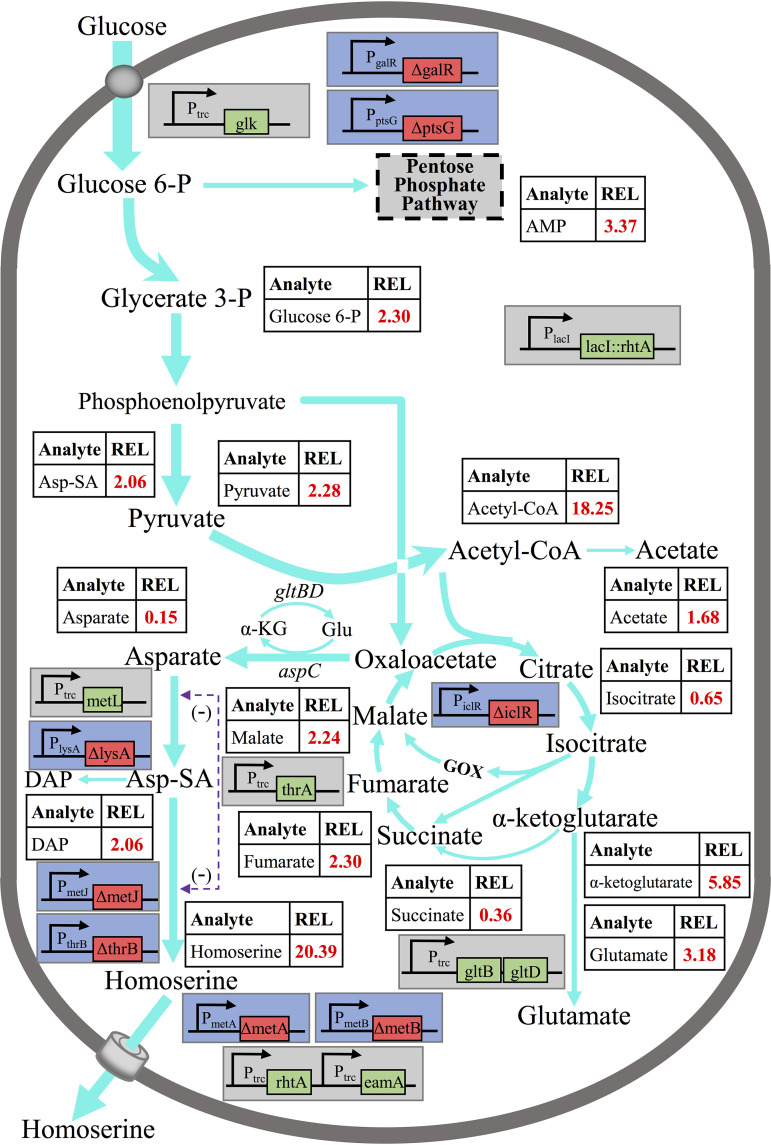
Systems metabolic engineering of E. coli for homoserine production. The data comprise relative concentrations of intracellular intermediates and metabolic flux predictions in the central metabolic pathways, as well as the genomic traits engineered into the strain. The thickness of the reaction arrows represents the predicted flux amount. The relative pool sizes of selected metabolites within two strains are given as REL values in the tables, with concentrations in E. coli W3110 taken as the reference for normalization between samples. The genomic traits include deletion of the transcriptional repressor (Δ*metJ*), deletion of *O*-succinylhomoserine lyase (Δ*metB*), overexpression of aspartate kinase (P*_trc_ metL*), deletion of homoserine kinase (Δ*thrB*), deletion of homoserine *O*-succinyltransferase (Δ*metA*), overexpression of homoserine dehydrogenase (P*_trc_ thrA*), deletion of diaminopimelate decarboxylase (Δ*lysA*), insertion of *rhtA* into the *lacI* locus (Δ*lacI*::*rhtA*), overexpression of the l-homoserine exporter (P*_trc_ rhtA*), overexpression of the cysteine/*O*-acetylserine exporter (P*_trc_ eamA*), deletion of the isocitrate lyase regulator (Δ*iclR*), deletion of the glucose-specific PTS enzyme IIBC component (Δ*ptsG*), deletion of the galactose operon repressor (Δ*galR*), overexpression of glucokinase (P*_trc_ glk*), and overexpression of the glutamate synthase operon (P*_trc_ gltBD*). REL, relative values; Glu, l-glutamate; α-KG, α-ketoglutarate; Asp-SA, l-aspartate 4-semialdehyde; DAP, diaminopimelic acid; GOX, glyoxylate cycle. The screened metabolomics data were subjected to PLS-DA. SCM (defined as follows: base VIP, >1.0; fold change, >1.2 or <0.8333; *q* < 0.05) were selected for subsequent chemical structure identification.

An intracellular-metabolite profiling study was performed in E. coli W3110 and HS33 to further investigate the effectiveness of the above-mentioned iterative genetic modifications and to identify important metabolic pathways that are closely associated with overproduction of l-homoserine (Fig. 8). In strain HS33, the intracellular levels of glycolytic intermediates, such as glucose-6-phosphate (G6P), pyruvate, and acetyl-CoA, increased 2.3-, 2.2-, and 18.2-fold, respectively. Additionally, a 3.3-fold increase in AMP was also detected in the strain. These results indicated that in strain HS33, disruption of the PTS (deletion of *ptsG*) effectively reduced consumption of PEP, which was beneficial in increasing the formation of precursor for l-homoserine biosynthesis. Moreover, the activation of a non-PTS by *glk* gene promoter replacement and *galR* gene deletion increased the consumption of ATP, resulting in the elevation of AMP. Also, 2.3- and 2.2-fold increases of fumarate and malate, as well as a 35.0% decrease in isocitrate, were observed in strain HS33, which indicated the activation of a glyoxylate shunt in the TCA cycle. The concentrations of α-ketoglutarate and l-glutamate increased 5.9- and 3.2-fold, respectively, in strain HS33. For two metabolites, aspartate and α-ketoglutarate, biosynthesis occurs through transamination between oxaloacetate and l-glutamate. Also, l-glutamate, as the donor of an α-amino for the biosynthesis of amino acids, can be synthesized from α-ketoglutarate by a transamination reaction. The increased l-glutamate and α-ketoglutarate pools indicated that the overexpression of the *gltBD* cluster could effectively provide an amino donor for the l-homoserine biosynthetic pathway and adjust the circulation of their pools within the proper range. With the increase of the l-homoserine pool, a decrease of aspartate was also observed. These results demonstrated that enhancement of the l-aspartate metabolism pathway associated with disruption of competitive pathways could effectively activate the biosynthesis of l-homoserine from l-aspartate. The concentration of the by-product 2,6-diaminopimelate (DAP) increased 2.2-fold due to knocking out *lysA*. Based on metabolome analyses, a better understanding is needed of the new limitations, such as carbon redirection and l-homoserine efflux, which would facilitate flux reinforcement by introducing an anaplerotic pathway or other regulatory mechanisms for more-efficient production of l-homoserine.

### Replenishment of oxaloacetate from pyruvate toward further improvement based on metabolomics analysis.

**FIG 9:**
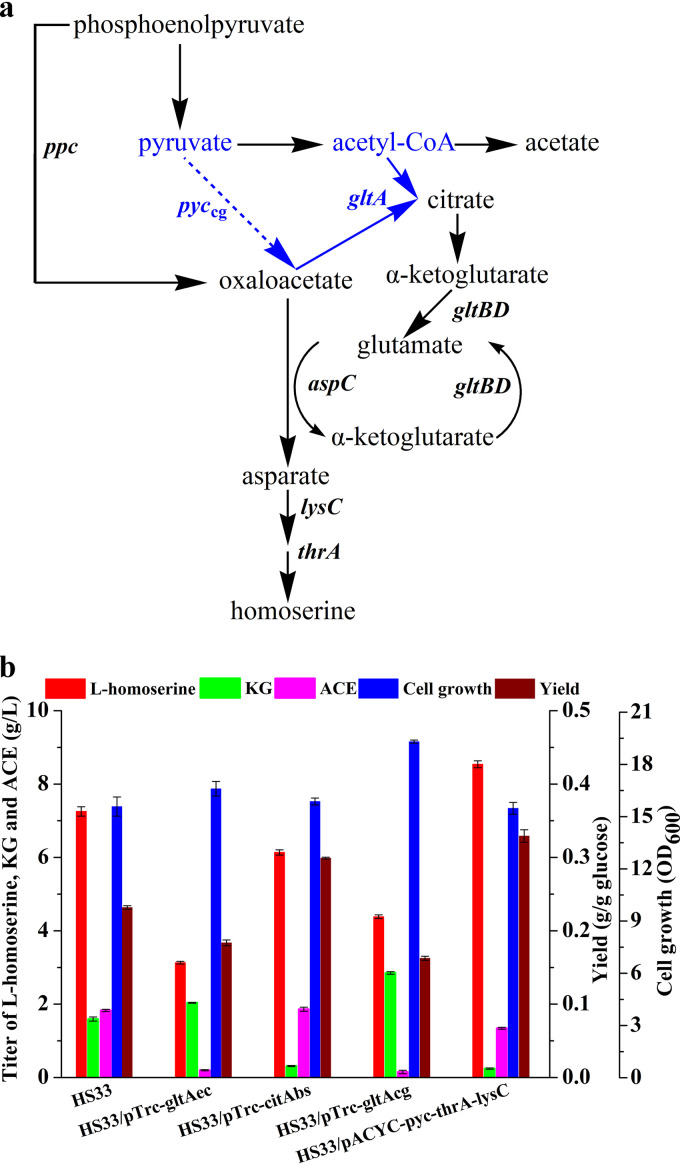
Introducing the anaplerotic route afforded by pyruvate carboxylase further improved the production of homoserine. (a) An overview of the central metabolic pathway in the strategy for diverting carbon to l-homoserine from acetyl-CoA. Blue arrows represent the paths (genes) introduced in the construction of a further improved strain. (b) The effects of overexpression of pyruvate carboxylase or citrate synthase on fermentation performance of l-homoserine. *gltA*_ec_, citrate synthase-encoding gene from E. coli; *citA*_bs_, encoding citrate synthase from Bacillus subtilis; *gltA*_cg_, citrate synthase-encoding gene from Corynebacterium glutamicum. The error bars represent SD.

While obtaining a global overview of metabolic variation, we found that intracellular levels of acetyl-CoA for strain HS33 were higher (more than 18-fold) than those for the wild-type strain, suggesting the carbon flux was not efficiently diverted to oxaloacetate or citrate for production. To address this new bottleneck, two strategies were investigated: (i) introducing the pyruvate carboxylase gene into the engineered strain to direct pyruvate to oxaloacetate rather than acetyl-CoA and (ii) introducing the citrate synthase gene into the engineered strain to direct acetyl-CoA to the TCA cycle. Since E. coli lacks the anaplerotic enzyme pyruvate carboxylase, pyruvate accumulation in the strain could not be directly altered by directing pyruvate to the aspartate branch (Fig. 9a). Although the *thrA* gene was previously overexpressed by replacing the *trc* promoter, the transcription level was increased by about 2-fold (see Fig. S3). To further enhance the flux of the l-aspartate branch, we overexpressed an aspartate-insensitive pyruvate carboxylase (encoded by *pyc*^P458S^) from C. glutamicum and threonine-insensitive bifunctional aspartokinase/homoserine dehydrogenase (encoded by *thrA*^G433R^) and aspartokinase (encoded by *lysC*) from E. coli in plasmid pACYC 184. Additionally, E. coli citrate synthase functions as a trimer of dimeric subunits, and its activity is allosterically inhibited by NADH and 2-oxoglutarate (36). In contrast, citrate synthase from Gram-positive bacteria and all eukaryotes is a simple dimer that is not allosterically regulated (37). Therefore, we overexpressed various citrate synthase genes, including *citA*_bs_ (Bacillus subtilis), *gltA*_cg_ (C. glutamicum), and *gltA*_ec_ (E. coli), in plasmid pTrc99A. Four plasmids were transformed into strain HS33, resulting in strain HS33/pACYC-*pyc*^P458S^-*thrA*^G433R^-*lysC*, HS33/pTrc-*citA*_bs_, HS33/pTrc-*gltA*_cg_, and HS33/pTrc-*gltA*_ec_ (Table 1).

**TABLE 1:** Bacterial strains and plasmids used in this study

Strain or plasmid	Characteristics	Source
Strains		
*E. coli* BL21	*E. coli* B F^−^ *dcm ompT hsdS*_B_(r_B_^−^ m_B_^−^) *gal* [*malB*^+^] K-12(λ S)	Trans BL21^*a*^
*E. coli* W3110	Wild type; F^−^ λ^−^ *IN* (*rrnD-rrnE*)*1 rph-1*	CGSC^*b*^
*E. coli* ΔJIB* Trc*metL*	W3110; Δ*metJ* Δ*metI* Δ*metB* Trc-*metL*	24
HS1	*E. coli* ΔJIB* Trc*metL*; Δ*thrB* Δ*metA* Trc-*thrA*	This study
HS2	HS1; Δ*lysA*	This study
HS3	HS2; Trc-*rhtA*	This study
HS4	HS2; Trc-*eamA*	This study
HS5	HS2; Δ*lacI*::Trc-*rhtA* Trc-*rhtA* Trc-*eamA*	This study
HS6	HS5; Δ*iclR*	This study
HS7	HS6; Δ*fumAC*	This study
HS8	HS7; Trc-*aspA*	This study
HS9	HS6; Δ*ptsG*	This study
HS10	HS6; Δ*ptsH*	This study
HS11	HS6; Δ*ptsI*	This study
HS12	HS6; Δ*crr*	This study
HS13	HS6; Δ*tktA*	This study
HS14	HS6; Δ*rpe*	This study
HS15	HS6; Δ*talB*	This study
HS16	HS6; Δ*zwf*	This study
HS17	HS6; Δ*ackA*	This study
HS18	HS6; Δ*pta*	This study
HS19	HS6; Δ*ilvA*	This study
HS20	HS6; Δ*poxB*	This study
HS21	HS6; Δ*argG*	This study
HS22	HS6; Δ*gadA*	This study
HS23	HS6; Δ*proB*	This study
HS24	HS6; Δ*crr ΔptsH*	This study
HS25	HS6; Δ*crr ΔptsI*	This study
HS26	HS6; Δ*crr ΔptsG*	This study
HS27	HS6; Δ*crr Δrpe*	This study
HS28	HS6; Δ*crr ΔtktA*	This study
HS29	HS9; Δ*galR* Trc-*glk*	This study
HS30	HS6; Trc-*gltB*	This study
HS31	HS30; Δ*argG*	This study
HS32	HS30; Δ*proB*	This study
HS33	HS9; Δ*galR* Trc-*glk* Trc-*gltB*	This study
Plasmids		
pTarget-X	A plasmid used to transcribe sgRNA targeting gene X in the genome. X refers to the genes in central metabolism and selected amino acid biosynthetic pathways.	This study
pACYC-*pyc*^P458S^-*thrA*^G433R^-*lysC*	Cm^r^; pACYC184 containing *pyc* (P458S), *thrA* (G433R), and *lysC*	This study
pTrc-gltA_ec_	pTrc99A containing *gltA* from *E. coli*	This study
pTrc-citA_bs_	pTrc99A containing *citA* from *B. subtilis*	This study
pTrc-gltA_cg_	pTrc99A containing *gltA* from *C. glutamicum*	This study

a TransGen Biotech, Shanghai, China.

b CGSC, E. coli Genetic Stock Center.

Five strains, including HS33, were cultivated in MS medium for 48 h. Among the tested strains, HS33/pACYC-*pyc*^P458S^-*thrA*^G433R^-*lysC* could produce 8.54 g/liter l-homoserine (0.33 g/g glucose), which was increased by 17.8% compared to strain HS33. Pyruvate carboxylase plays an anaplerotic role for carbon redistribution by directing pyruvate to the aspartate branch, which was shown to be efficient in reducing the accumulation of α-ketoglutarate (0.26 g/liter) and acetate (1.35 g/liter). However, the other three strains that overexpressed citrate synthase produced smaller amounts of l-homoserine than the corresponding strain, HS33 (Fig. 9b). The overexpression of citrate synthase by B. subtilis resulted in reducing the accumulation of α-ketoglutarate, but the l-homoserine yield was higher than that of the control (0.30 versus 0.23 g/g glucose). In contrast, the overexpression of citrate synthase by C. glutamicum and E. coli resulted in improved growth and reduced by-product (acetate) accumulation. However, α-ketoglutarate accumulated significantly, which was probably due to the fact that the flux was altered by the enzyme by directing oxaloacetate to the citrate branch.

### Fed-batch production of l-homoserine.

**FIG 10:**
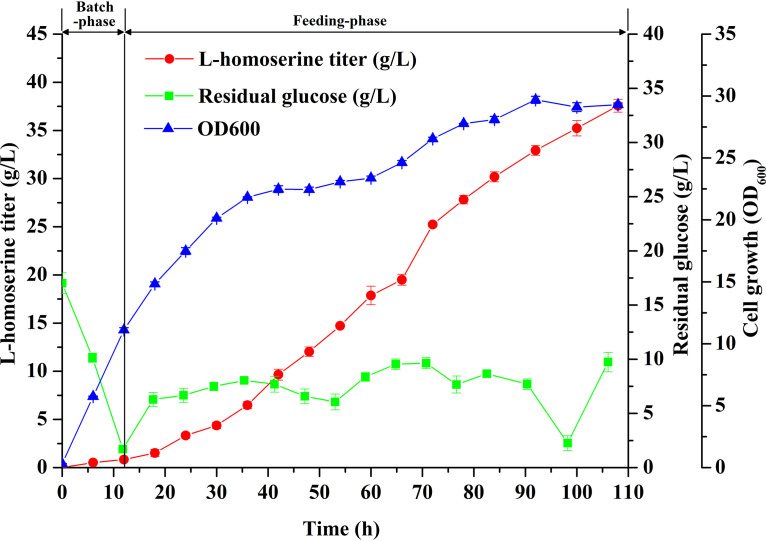
Fed-batch fermentation profile of HS33/pACYC-*pyc*^P458S^-*thrA*^G433R^-*lysC* in a 5-liter bioreactor. Time profiles of l-homoserine production, glucose concentrations, and cell growth during the fed-batch cultivation are shown. The error bars represent SD.

In order to scale up for l-homoserine production by HS33/pACYC-*pyc*^P458S^-*thrA*^G433R^-*lysC* from glucose, fed-batch culture was implemented in a 5-liter bioreactor containing 2 liters of medium. Cell growth, l-homoserine production, and residual glucose during the fed-batch culture are shown in Fig. 10. A total of 450 ml feeding medium containing 500 g/liter glucose was added to the bioreactor during the cultivation, and the residual glucose concentration was maintained below 10 g/liter. The cell growth entered stationary phase within 40 h, and the optical density at 600 nm (OD_600_) fluctuated around 30 till the end of cultivation. Within 108 h of the fermentation, l-homoserine production continuously increased and was independent of cell growth, and the maximum titer achieved was 37.57 g/liter with a yield of 0.31 g/g glucose, representing a 4.4-fold increase compared to the titer achieved in flask shake cultivation. By-products of amino acids were almost undetectable. However, further effort is needed to increase the titer and shorten the fermentation time, such as optimizing the expression of the recognized genes and the culture conditions.

## DISCUSSION

This work showed the construction of a basic homoserine producer through a combination of the traditional pull-push-block effects of metabolic engineering and rational genetic modifications. In accordance with the results reported previously, the disruption of competitive and degradative pathways (l-threonine, l-lysine, and l-methionine pathways) contributed to increasing the production of l-homoserine (13). When it comes to the accumulation of target products that are toxic to cells or that inhibit the activity of enzymes in the metabolic pathway, strengthening the exportation of these metabolites is of great importance to relieve the cell burden and enhance production (32, 38–40). Here, strengthening the transport system and constructing a constitutive expression system not only allowed constitutive expression of modified genes without the addition of inducer, it also avoided suppressing cell growth caused by the inducer and l-homoserine (32). Additionally, observations after deleting the transcriptional regulator IclR indicated that the TCA cycle could operate smoothly when activating the glyoxylate shunt flux. However, the diversion of flux away from forming oxaloacetate from fumaric acid by deletion of fumarase genes or overexpression of *aspA* dramatically reduced the production of l-homoserine, which may be attributed to the toxicity of fumaric acids (41).

In addition to the above-mentioned rather obvious genetic modifications, we also tested the impacts of another 39 different genes on l-homoserine production by using CRISPRi-based gene repression methodology. The results of gene repression indicated that use of the CRISPRi system was a feasible method to regulate gene expression with less labor and time consumption. Specifically, downregulation of the PTS is widely approved for reinforcing the biosynthesis of PEP-derived products (42, 43). The repression of *zwf*, *tktA*, *rpe*, and *talB* disrupted the pentose phosphate pathway, which can direct more carbon to the synthesis of precursors in the glycolytic pathway. *argA*-, *argG*-, *proB*-, and *gadA*-repressing regulation may contribute to accumulating l-glutamate and l-aspartate and therefore provide more precursors for l-homoserine biosynthesis (44). Also, the repression of *ackA*, *pta*, and *poxB* contributed to reducing secretion of acetate, which blocked the by-product formation pathway and released acid stress. Moreover, validation of screened candidate genes by individual gene deletion further indicated that sequence-specific control of gene expression on a genome-wide scale is a practical tool for engineering gene-regulatory systems in l-homoserine biosynthesis (45). However, we observed that deletions of some genes led to a decrease in l-homoserine production, which was not consistent with the results showing that these genes were repressed by CRISPR-dCas9. The possibility of completely inhibiting gene expression with CRISPRi is low, and in some cases, even a low expression level of certain genes located in metabolic pathways could potentially contribute enough enzyme activity to maintain its flux and thus cover the effect of targeting such genes (46).

Based on CRISPRi-based systematic analysis, integration of glucose uptake and recovery of l-glutamate were adopted to develop a stable strain with better performance. In the metabolism of the carbon skeletons of l-homoserine, PTS disruption is beneficial to improve the size of the intracellular pool of PEP, which is a direct precursor of l-aspartate family amino acids (42). However, the observed phenotype of PTS disruption mutations other than *ptsG* mutation was in accord with the reports that low growth rates were observed as the strains would consume ATP for phosphorylation of glucose through a non-PTS system (47). Therefore, restoration of glucose transport capacity could efficiently improve l-homoserine production. In terms of engineering amino acid supply pathways, enhancing circulation of amino donors by converting α-ketoglutarate to l-glutamate could also efficiently enhance l-homoserine production. However, increasing the availability of the intracellular amino acids l-aspartate and l-glutamate by blocking the metabolic pathways failed to increase the accumulation of l-homoserine. The results indicated that the regulation of l-aspartate and l-glutamate metabolism is complicated, as they are precursors involved in more than 10 pathways, which made it difficult to identify the major bottleneck for l-homoserine accumulation.

Metabolomics analysis revealed that the multiplex design of the metabolic network caused less flexibility of flux distribution on the metabolome level. The significant variations of strain HS33 directed more carbon toward l-homoserine biosynthesis after the multiple genetic modifications. Additionally, they facilitated understanding of the underlying metabolic effects and provided relevant targets for strain modification to achieve a preferable performance. The results demonstrated that introducing the anaplerotic pathway to direct pyruvate to oxaloacetate was superior to the strategy of directing acetyl-CoA to the TCA cycle. The metabolic bottleneck for the production of l-homoserine could be efficiently relieved by modification of metabolic flux at both the oxaloacetate and pyruvate nodes. Directing pyruvate to the l-aspartate branch could directly realize carbon redistribution, while regulation of the TCA cycle was quite complex, as it is a crucial component of metabolism in organisms (48).

In conclusion, 5 levels of bottlenecks, i.e., the transport system, transcriptional regulation, carbon utilization, amino donor circulation, and the anaplerotic path, that sequentially limited l-homoserine biosynthesis were identified and resolved after systematic searching and multiplex design of the l-homoserine biosynthesis pathway. The l-homoserine production of the optimal strain HS33/pACYC-*pyc*^P458S^-*thrA*^G433R^-*lysC* reached 8.54 g/liter (0.33 g/g glucose, 62.4% of the maximum theoretical yield) and 37.57 g/liter (0.31 g/g glucose, 58.6% of the maximum theoretical yield) in shake flask cultivation and fed-batch fermentation without optimizing the expression of the selected genes or the culture conditions (Table 2). Further, it is expected that l-homoserine production by the optimal strain constructed in this study can be further enhanced by optimizing the expression of the positive genes and the fermentation process, which would unlock the potential of the strain for the production of l-homoserine and its derivatives on a large scale.

**TABLE 2:** l-Homoserine production and relevant fermentation parameters of the engineered strains

Strain	l-Homoserine titer (g/liter) (±SD)	Cell growth (OD_600_)	Cultivation mode	Analytical method^*a*^	Source
*E. coli* NZ10	10.6		Shake flask (glucose)	HPLC (*o*-phthalaldehyde)	14
*C. glutamicum* 9366-EMS	14.5		Batch cultivation (sucrose)	Amino analyzer	53
*C. glutamicum* MH20-22B	3.0		Shake flask (glucose)	HPLC (phenylisothiocyanate)	12
HM5(pBRmetL–pNrhtA)	39.54 ± 1.10	38.99 ± 0.55	Fed-batch (glucose)	HPLC (*o*-phthalaldehyde)	13
HS1	1.85 ± 0.11	14.01 ± 0.14	Shake flask (glucose)	Amino analyzer	This study
HS2	2.01 ± 0.11	15.21 ± 0.11	Shake flask (glucose)	Amino analyzer	This study
HS5	3.14 ± 0.01	15.17 ± 0.27	Shake flask (glucose)	Amino analyzer	This study
HS6	3.26 ± 0.13	14.67 ± 0.44	Shake flask (glucose)	Amino analyzer	This study
HS9	5.28 ± 0.10	17.65 ± 0.27	Shake flask (glucose)	Amino analyzer	This study
HS12	5.52 ± 0.04	12.06 ± 0.47	Shake flask (glucose)	Amino analyzer	This study
HS29	6.27 ± 0.05	17.32 ± 0.44	Shake flask (glucose)	Amino analyzer	This study
HS30	3.95 ± 0.07	17.07 ± 0.35	Shake flask (glucose)	Amino analyzer	This study
HS33	7.25 ± 0.20	15.58 ± 0.55	Shake flask (glucose)	Amino analyzer	This study
HS33/pACYC-*pyc*^P458S^-*thrA*^G433R^-*lysC*	8.54 ± 0.10	15.48 ± 0.34	Shake flask (glucose)	Amino analyzer	This study
HS33/pACYC-*pyc*^P458S^-*thrA*^G433R^-*lysC*	37.57 ± 0.66	29.3 ± 0.14	Fed-batch (glucose)	Amino analyzer	This study

a HPLC, high-performance liquid chromatography.

## MATERIALS AND METHODS

### Strains, plasmids, and culture conditions.

All the strains and plasmids used in this study are listed in Table 1. E. coli BL21 was used for plasmid construction, and the OSH-producing strain (24) was used as the original strain. The low-copy-number plasmid pACYC184 was used as the backbone for overexpressing the mutated *thrA* gene (G433R) and *lysC* gene from E. coli W3110 and the mutated *pyc* gene (P458S) from C. glutamicum ATCC 13032. Plasmid pTrc99A was used for overexpressing citrate synthase genes from E. coli W3110, B. subtilis 168, and C. glutamicum ATCC 13032. Luria-Bertani medium (10 g/liter tryptone, 5 g/liter yeast extract, and 10 g/liter NaCl) was used for cell cultivation. When needed, kanamycin and chloramphenicol were supplemented at final concentrations of 50 μg/ml.

### Plasmid construction.

To construct plasmid pACYC-*pyc*^P458S^-*thrA*^G433R^-*lysC*, the mutated *pyc* gene (P458S) (NCBI gene ID 3344537) was amplified from C. glutamicum, and the *thrA* gene (G433R) and *lysC* gene were amplified from E. coli (49). The three fragments were ligated into pACYC184 by the standard protocol of Gibson assembly (50).

For expression of citrate synthase, the corresponding genes, *gltA*_ec_ (NCBI gene ID 12930947), *citA*_bs_ (NCBI gene ID 936270), and *gltA*_cg_ (NCBI gene ID 3344398), were amplified from the genomic DNA of E. coli, B. subtilis, and C. glutamicum using the primers GltA_ec__F/GltA_ec__R, CitA_bs__F/CitA_bs__R, and GltA_cg__F/GltA_cg__R. Then, the three fragments were assembled with the pTrc99A backbone fragment obtained using the primers pTrc99A-F/pTrc99A-R to yield pTrc-*gltA*_ec_, pTrc-*citA*_bs_, and pTrc-*gltA*_cg_. The primers used for plasmid construction are listed in Table 3.

**TABLE 3:** Primers used for plasmid construction

Primer	Sequence (5′–3′)	Source
pACYC-F	CCTGATGAATGCTCATCCGG	This study
pACYC-R	GCAAATATTATACGCAAGG	This study
Pyc-F	CCTTGCGTATAATATTTGCATGTCGACTCACACATCTTC	This study
Pyc-R	ACACTCGCATGTATATCTCCTTTTAGGAAACGACGACGATCA	This study
ThrA-F	AAGGAGATATACATGCGAGTGTTGAAGTTCGG	This study
ThrA-R	TTTCAGACATGTATATCTCCTTTTAGACTCCTAACTTCCATG	This study
LysC-F	AGGAGTCTGAAAGGAGATATACATGTCTGAAATTGTTGTCTC	This study
LysC-R	CCTGATGAATGCTCATCCGGTTACTCAAACAAATTACTAT	This study
pTrc99A-F	TCTGTTTCCTGTGTGAAATT	This study
pTrc99A-R	TCTAGAGTCGACCTGCAG	This study
GltA_ec__F	AATTTCACACAGGAAACAGAATGGCTGATACAAAAGCAAA	This study
GltA_ec__R	GCCTGCAGGTCGACTCTAGATTAACGCTTGATATCGCTTT	This study
CitA_bs__F	AATTTCACACAGGAAACAGAATGGTACATTACGGATTAAA	This study
CitA_bs__R	GCCTGCAGGTCGACTCTAGATTATGAAAGAACTTCCTCGG	This study
GltA_cg__F	AATTTCACACAGGAAACAGAATGTTTGAAAGGGATATCGT	This study
GltA_cg__R	GCCTGCAGGTCGACTCTAGATTAGCGCTCCTCGCGAGGAA	This study

### Construction of an sgRNA-expressing plasmid for the CRISPRi system.

The pdCas9 plasmid with a catalytically dead Cas9 mutant was stored in our collection (45). The original plasmid, pCas, and pTargetF, containing the Cas9 gene (51) and sgRNA, were donated by Sheng Yang from the Institute of Plant Physiology and Ecology (Chinese Academy of Sciences, Shanghai, China). The sgRNA-expressing plasmid library targeting selected genes was constructed according to previously reported methods (22). Briefly, sgRNA cassettes were generated through PCR-based site-directed mutagenesis using the original pTarget as a template. Primer sequences used for generation of sgRNA cassettes and the corresponding sgRNA expression vectors are listed in Table 4. Every sgRNA-expressing plasmid for downregulation of target genes was transformed into electrocompetent cells containing plasmid pdCas9 to obtain a series of strains: HS6(pdCas9, pTarget-X), where X refers to the genes in the central metabolism and selected amino acid biosynthetic pathways.

**TABLE 4:** Primers used for CRISPRi

Primer^*a*^	Target	Sequence (5′–3′)^*c*^
pTarget-ptsH	*ptsH*	AT*ACTAGT***GGTGTGCAGACCGTTCGGAG**GTTTTAGAGCTAGAAATAGC
pTarget-ptsG	*ptsG*	AT*ACTAGT***GCGATTTACCGACCTTTTGC**GTTTTAGAGCTAGAAATAGC
pTarget-ptsI	*ptsI*	AT*ACTAGT***CGGGTATCGCTTTCGGTAAA**GTTTTAGAGCTAGAAATAGC
pTarget-crr	*crr*	AT*ACTAGT***TCCGGTATCCTTCTTGTCGT**GTTTTAGAGCTAGAAATAGC
pTarget-tktA	*tktA*	AT*ACTAGT***ATGCTATTCGTGCGCTGAGC**GTTTTAGAGCTAGAAATAGC
pTarget-tktB	*tktB*	AT*ACTAGT***ATGCGATTCGCGCACTCAGT**GTTTTAGAGCTAGAAATAGC
pTarget-rpe	*rpe*	AT*ACTAGT***CTCAATTCTGTCGGCTGATT**GTTTTAGAGCTAGAAATAGC
pTarget-rpiA	*rpiA*	AT*ACTAGT***CCTACACCAACAATGGTGCC**GTTTTAGAGCTAGAAATAGC
pTarget-rpiB	*rpiB*	AT*ACTAGT***ATCAGTACGCTCTGACGACC**GTTTTAGAGCTAGAAATAGC
pTarget-talA	*talA*	AT*ACTAGT***CTGTCGTGGCAGACAGCGGC**GTTTTAGAGCTAGAAATAGC
pTarget-talB	*talB*	AT*ACTAGT***TTCGTCAGTACACCACCGTA**GTTTTAGAGCTAGAAATAGC
pTarget-zwf	*zwf*	AT*ACTAGT***GGTCATTTTCGGCGCGAAAG**GTTTTAGAGCTAGAAATAGC
pTarget-ackA	*ackA*	AT*ACTAGT***TCATCGATGCAGTAAATGGT**GTTTTAGAGCTAGAAATAGC
pTarget-pta	*pta*	AT*ACTAGT***TACCGGAACCAGCGTCGGTC**GTTTTAGAGCTAGAAATAGC
pTarget-poxB	*poxB*	AT*ACTAGT***CCCTGCCGATTCGAGTGTTT**GTTTTAGAGCTAGAAATAGC
pTarget-ldhA	*ldhA*	AT*ACTAGT***GTACTGTTTTGTGCTATAAA**GTTTTAGAGCTAGAAATAGC
pTarget-dld	*dld*	AT*ACTAGT***ACGAGCAAGTTCATTCAAAA**GTTTTAGAGCTAGAAATAGC
pTarget-adhE	*adhE*	AT*ACTAGT***GAAACTGGCATATTCACGCT**GTTTTAGAGCTAGAAATAGC
pTarget-mdh	*mdh*	AT*ACTAGT***CAATACCGCCAGCAGCGCCG**GTTTTAGAGCTAGAAATAGC
pTarget-serA	*serA*	AT*ACTAGT***GAAGGCTTTCCAGCGCCTTT**GTTTTAGAGCTAGAAATAGC
pTarget-pck	*pck*	AT*ACTAGT***ATAAGCCTCGAGTTCTTGCG**GTTTTAGAGCTAGAAATAGC
pTarget-pps	*pps*	AT*ACTAGT***TGGTTATACCAAAGCACCAG**GTTTTAGAGCTAGAAATAGC
pTarget-maeB	*maeB*	AT*ACTAGT***TCCCTGGAACTGGAAATTCA**GTTTTAGAGCTAGAAATAGC
pTarget-ilvA	*ilvA*	AT*ACTAGT***CCTTCCGGAGCACCGGACAG**GTTTTAGAGCTAGAAATAGC
pTarget-tdcB	*tdcB*	AT*ACTAGT***ATAATGTCATCAATAGCAAC**GTTTTAGAGCTAGAAATAGC
pTarget-pyrL	*pyrL*	AT*ACTAGT***CCAGCGTCTTTTTTCAGACG**GTTTTAGAGCTAGAAATAGC
pTarget-pyrB	*pyrB*	AT*ACTAGT***GATTAAGGTCATCGCGACTA**GTTTTAGAGCTAGAAATAGC
pTarget-asnA	*asnA*	AT*ACTAGT***ACGTTGTTTGGCAATGTAAG**GTTTTAGAGCTAGAAATAGC
pTarget-asnB	*asnB*	AT*ACTAGT***CATCAGGCGTGACAGCTCGA**GTTTTAGAGCTAGAAATAGC
pTarget-purA	*purA*	AT*ACTAGT***TTTACCTTCGTCACCCCATT**GTTTTAGAGCTAGAAATAGC
pTarget-putA	*putA*	AT*ACTAGT***GTCGTCCAGCTTAACCCCCA**GTTTTAGAGCTAGAAATAGC
pTarget-argG	*argG*	AT*ACTAGT***ATACCAATACGTTGACCTAC**GTTTTAGAGCTAGAAATAGC
pTarget-glnA	*glnA*	AT*ACTAGT***CATCGTCAGTACGTGTTCAG**GTTTTAGAGCTAGAAATAGC
pTarget-ltaE	*ltaE*	AT*ACTAGT***TCATCGCTTCGAGCATGGCG**GTTTTAGAGCTAGAAATAGC
pTarget-tdh	*tdh*	AT*ACTAGT***GCCCTCTTCCGCTTTCAGTT**GTTTTAGAGCTAGAAATAGC
pTarget-argA	*argA*	AT*ACTAGT***TGATATAGGGAACCGAATGG**GTTTTAGAGCTAGAAATAGC
pTarget-proB	*proB*	AT*ACTAGT***CGAGTTTTACCACCAGCGTC**GTTTTAGAGCTAGAAATAGC
pTarget-aspA	*aspA*	AT*ACTAGT***ACACCATAGTAGGCATCAGC**GTTTTAGAGCTAGAAATAGC
pTarget-gadA	*gadA*	AT*ACTAGT***GTGAATCGAGTAGTTCTGAG**GTTTTAGAGCTAGAAATAGC
pTarget-null^*b*^		AT*ACTAGT*GTTTTAGAGCTAGAAATAGC
pTarget R-common		ACTAGTATTATACCTAGGACTGAGC

a Each pTarget primer has the same reverse primer, pTarget R-common (listed last).

b pTarget-null represents the primer used to construct pTarget-null without the N20 sequence, used as a negative control.

c The italic letters represent the restriction enzyme cutting site (SpeI), and the boldface letters represent the 20-bp region complementary to the targeting region of the selected gene.

### Genetic manipulation of E. coli derivatives using CRISPR-Cas9.

Gene insertion and deletion and chromosomal promoter replacement were achieved by using the CRISPR-Cas9 system, as previously described (24). The detailed protocol of gene editing is described in the supplemental material. The primers used in this study are listed in Table 5. Electrocompetent cells were prepared as previously described (51).

**TABLE 5:** Primers used for genome editing

Purpose	Target	Primer^*a*^	Sequence (5′–3′)
Gene knockout	*metA*	pTarget-*metA*	TAATACTAGTTCTGGCAGAGACGGAAGAAGGTTTTAGAGCTAGAAATAGC
		L-*metA*-F	ATGAGAAAAAATCGCCTACGCCCCC
		L-*metA*-R	TCACAGAAGAAACCTGATTACCTCACTACA
		R-*metA*-F	TAATCAGGTTTCTTCTGTGATAGTCGATCG
		R-*metA*-R	ATAAAGACTTTCACATTGGCGTTGA
		T-*metA*-F	TGCTTAAGCAGAAATAATCG
		T-*metA*-R	GTAAATTTTGCCTGCTTCAT
	*lysA*	pTarget-*lysA*	TAATACTAGTACGATGCGCAAATTATTCGTGTTTTAGAGCTAGAAATAGC
		L-*lysA*-F	CGTTCTGTTCCCGCAGGCGTATGGA
		L-*lysA*-R	CTAACCGCAGAACAAACTCCAGATAAGTGC
		R-*lysA*-F	GGAGTTTGTTCTGCGGTTAGTCGCTGGTTG
		R-*lysA*-R	ATCCTGCCTGGCTTTGAAAACCGTT
		T-*lysA*-F	ATAGCTGTCAGTACGGGAAA
		T-*lysA*-R	TTACCACAACGAACAAAAAG
	*ΔlacI::Trc-rhtA*	pTarget-*lacI*	GCTCTAAAACAATGAGTGAGCTAACTCACAACTAGTATTATACCTAGGAC
		L-*lacI*-F	AAGTGGTATGGCCGACAGAT
		L-*lacI*-R	TAATTGTCAAATTCACCACCCTGAATTGAC
		*lacI*::Trc-*metA*-F	GGTGGTGAATTTGACAATTAATCATCCGGC
		*lacI*::Trc-*metA*-R	AAGTGTAAAGTTAATTAATGTCTAATTCTT
		R-*lacI*-F	CATTAATTAACTTTACACTTTATGCTTCCG
		R-*lacI*-R	GGTCAAATTCAGACGGCAAA
		T-*lacI*-F	ATGATAGTTTTCGACTGACC
		T-*lacI*-R	AAAAGATAATTCATCCCACC
	*iclR*	pTarget-*iclR*	TAATACTAGTCTAACCACGATGCAACAGCAGTTTTAGAGCTAGAAATAGC
		L-*iclR*-F	GATCGTTGACGTCCGGAGAAATAGA
		L-*iclR*-R	CCAGAAAAAGGACAGTCTCTTTTTTCTGTA
		R-*iclR*-F	AGAGACTGTCCTTTTTCTGGCGGGCAGAGG
		R-*iclR*-R	AGTGTCGTGTTTTCGTTTTTATTAA
		T-*iclR*-F	GGTTTCAATCAGGATCAGAT
		T-*iclR*-R	TCGGCATACTATACGGTTTT
	*fumB*	pTarget-*fumB*	GCTCTAAAACAATAGCTTGTGTTGCTCAAGACTAGTATTATACCTAGGAC
		L-*fumB*-F	TTATTCAGATGTTTATGCTGCTGAC
		L-*fumB*-R	GAAGAGGTTACATAGCTTCCAGCCTGTAAC
		R-*fumB*-F	GGAAGCTATGTAACCTCTTCGGCCCAGCGC
		R-*fumB*-R	CCCACCACTCGATTAATTCATACAT
		T-*fumB*-F	CAGAAATTCATCTCCGTACC
		T-*fumB*-R	ACCGGATAAGGAATTCACGC
	*fumAC*	pTarget-*fumAC*	TAATACTAGTGATGCCTGTAAAAAACACGGGTTTTAGAGCTAGAAATAGC
		L-*fumAC*-F	TTGTGGTTAGGATCTTTATAGTTAC
		L-*fumAC*-R	TTGCAGATTACATTGTTCTCTCACTTACTG
		R-*fumAC*-F	GAGAACAATGTAATCTGCAACATACAGGTG
		R-*fumAC*-R	CCAGGTCGATAACCTTTCGCAAGCA
		T-*fumAC*-F	ATTTTTCTTCACCCTGCATA
		T-*fumAC*-R	CTTGAGCAACACAAGCTATT
	*ptsG*	pTarget-*ptsG*	ATACTAGTGCGATTTACCGACCTTTTGCGTTTTAGAGCTAGAAATAGC
		L-*ptsG*-F	TGGATCTCGGATTTTACATC
		L-*ptsG*-R	TTACGGATTACATAATTGAGAGTGCTCCTG
		R-*ptsG*-F	CTCAATTATGTAATCCGTAAGACGTTGGGG
		R-*ptsG*-R	CTCAATTATGTAATCCGTAAGACGTTGGGG
		T-*ptsG*-F	TATTGAGCAGGATAATGTCG
		T-*ptsG*-R	ACTAAAGTACGTCAGCAAGA
	*ptsH*	pTarget-*ptsH*	ATACTAGTGGTGTGCAGACCGTTCGGAGGTTTTAGAGCTAGAAATAGC
		L-*ptsH*-F	GAAGGTATTCTTGCAGGTAT
		L-*ptsH*-R	CGGGAAATTACATTGTATTTCCCCAACTTA
		R-*ptsH*-F	AAATACAATGTAATTTCCCGGGTTCTTTTA
		R-*ptsH*-R	TAGCAGGTAGTTCCAGAGAA
		T-*ptsH*-F	TGGCAGGTGAAGAGATTAAA
		T-*ptsH*-R	TTGGTTGGATTGACGTAAAC
	*ptsI*	pTarget-*ptsI*	ATACTAGTCGGGTATCGCTTTCGGTAAAGTTTTAGAGCTAGAAATAGC
		L-*ptsI*-F	AAAGCCAAAGCTGAATCGAT
		L-*ptsI*-R	TCGTGGATTACATAACCCTACCTTACTTGT
		R-*ptsI*-F	TAGGGTTATGTAATCCACGAGATGCGGCCC
		R-*ptsI*-R	TCCATGTTGGAGATAACAAC
		T-*ptsI*-F	AAAGCACCTTTTTAGGTGCT
		T-*ptsI*-R	GAGCGATGAATTGATTTTGC
	*crr*	pTarget-*crr*	ATACTAGTTCCGGTATCCTTCTTGTCGTGTTTTAGAGCTAGAAATAGC
		L-*crr*-F	GCAAAGAGATCGAAATCTAC
		L-*crr*-R	GCAAGAATTACATGATCTTCTCCTAAGCAG
		R-*crr*-F	GAAGATCATGTAATTCTTGCCGCAGTGAAA
		R-*crr*-R	AAATCAAAATCCTTGCCGAG
		T-*crr*-F	ATACATGAACTTCCCGAAAG
		T-*crr*-R	CATTATGACACTTTCTACGG
	*galR*	pTarget-*galR*	TAATACTAGTGGCGAACCAGACGAAAGCGGGTTTTAGAGCTAGAAATAGC
		L-*galR*-F	CTCTGATTCAGTAAAAGCGA
		L-*galR*-R	TCTGCGGTTACATGAAAATACCTTAGTGGG
		R-*galR*-F	TAACCGCAGAACAAACTCCAGATAAGTGCT
		R-*galR*-R	TTCAAGTAGCGGTGATTCCT
		T-*galR*-F	GATTAGGCGTAATTAGAACG
		T-*galR*-R	TAATTCGGTACGTTCTGTTC
	*tktA*	pTarget-*tktA*	GCACTAGTGCTTCCGGGCTGCAAACAAGGTTTTAGAGCTAGAAATAGC
		L-*tktA*-F	TTGTCAGACGTTTAGCGTAT
		L-*tktA*-R	ATGCTAATTACATTTTGACTCCAGATCGGA
		R-*tktA*-F	AGTCAAAATGTAATTAGCATTTCGGGTAAA
		R-*tktA*-R	CGAGAATATAATAAGGGCCA
		T-*tktA*-F	ATCCGTCATCATATCCATCA
		T-*tktA*-R	AGAGAGTCCGATGAGTAATT
	*rpe*	pTarget-*rpe*	ATACTAGTCTCAATTCTGTCGGCTGATTGTTTTAGAGCTAGAAATAGC
		L-*rpe*-F	TATATTTGAACCGCTACGGT
		L-*rpe*-R	TTCAAACTTACATCCGCTTCTCCTTGAGAA
		R-*rpe*-F	GAAGCGGATGTAAGTTTGAAGATATTCGCG
		R-*rpe*-R	GACAAACAGCATCTGTTGTG
		T-*rpe*-F	CGACTTTTCTCGTTATATCC
		T-*rpe*-R	GGCAGAAGGTCATTTATAGA
	*talB*	pTarget-*talB*	ATACTAGTTTCGTCAGTACACCACCGTAGTTTTAGAGCTAGAAATAGC
		L-*talB*-F	ACTGGTACACAATGACTGAA
		L-*talB*-R	AGAATGATTACATGATAGTATTTCTCTTTA
		R-*talB*-F	TACTATCATGTAATCATTCTTAGCGTGACC
		R-*talB*-R	TTCCAGCGTCTCTTTAATAG
		T-*talB*-F	ATAAAGTTGTGCCAGAGAAC
		T-*talB*-R	TCTGAATGCTGCAACCACTT
	*zwf*	pTarget-*zwf*	ATACTAGTGGTCATTTTCGGCGCGAAAGGTTTTAGAGCTAGAAATAGC
		L-*zwf*-F	TCTGTGCCAGATGAAGTTTA
		L-*zwf*-R	GCAGATATTACATGTCATTCTCCTTAAGTT
		R-*zwf*-F	GAATGACATGTAATATCTGCGCTTATCCTT
		R-*zwf*-R	ATGTCGTTATAGGAGGTGAT
		T-*zwf*-F	TTTATCCAGTGAATGACGGA
		T-*zwf*-R	AACAGAGCACCATCAAACAT
	*ackA*	pTarget-*ackA*	ATACTAGTTCATCGATGCAGTAAATGGTGTTTTAGAGCTAGAAATAGC
		L-*ackA*-F	CATAAAACGGATCGCATAAC
		L-*ackA*-R	TGTGAAATCACATGGAAGTACCTATAATTG
		R-*ackA*-F	TACTTCCATGTGATTTCACACCGCCAGCTC
		R-*ackA*-R	TGATGTTGGTGTTTTTGGCA
		T-*ackA*-F	CTTCATAAAACCAGTTAAGG
		T-*ackA*-R	ACTTTAGCTTTGGAAGAGTC
	*pta*	pTarget-*pta*	ATACTAGTTACCGGAACCAGCGTCGGTCGTTTTAGAGCTAGAAATAGC
		L-*pta*-F	TAAATGCGTTGACACCTCTA
		L-*pta*-R	GACGAGATTACACGGTTTATCCTCTTTCGT
		R-*pta*-F	ATAAACCGTGTAATCTCGTCATCATCCGCA
		R-*pta*-R	TACCGTTATCAATGGTTCCT
		T-*pta*-F	CCTTACAACCTGTACAAAGA
		T-*pta*-R	ATTTGCGTGGCAATATAGGT
	*ilvA*	pTarget-*ilvA*	ATACTAGTCCTTCCGGAGCACCGGACAGGTTTTAGAGCTAGAAATAGC
		L-*ilvA*-F	TACGGTAACTTTGCGGAAAA
		L-*ilvA*-R	TTTTTCCCTACATTATTAACCCCCCAGTTT
		R-*ilvA*-F	GTTAATAATGTAGGGAAAAATGCCTGATAG
		R-*ilvA*-R	GAGATTAAACTTACTACTGG
		T-*ilvA*-F	AAACGCTGGAACAATACGAC
		T-*ilvA*-R	AAACGCTGTTGCAGTATCAG
	*poxB*	pTarget-*poxB*	ATACTAGTCCCTGCCGATTCGAGTGTTTGTTTTAGAGCTAGAAATAGC
		L-*poxB*-F	ATAACGTTACCGAAGGCTTT
		L-*poxB*-R	ACCCTTTTTACATGGTTCTCCATCTCCTGA
		R-*poxB*-F	GAGAACCATGTAAAAAGGGTGGCATTTCCC
		R-*poxB*-R	AATTCCCATGCTTCTTTCAG
		T-*poxB*-F	AGATAAATTCCTGTTGCTGG
		T-*poxB*-R	CAAATGGTGAACGAATCACA
	*argG*	pTarget-*argG*	ATACTAGTATACCAATACGTTGACCTACGTTTTAGAGCTAGAAATAGC
		L-*argG*-F	TTTCAAATCCCACTACGAAG
		L-*argG*-R	TGTCGAATTACATAAAATAACACCCTGCTT
		R-*argG*-F	TTATTTTATGTAATTCGACATCAACCCTGC
		R-*argG*-R	TCTTCATTTAAATGGAAGCC
		T-*argG*-F	AAAGCCCAGTTATTCTGTAG
		T-*argG*-R	GCCATGGAAACAGTTTATGT
	*gadA*	pTarget-*gadA*	ATACTAGTGTGAATCGAGTAGTTCTGAGGTTTTAGAGCTAGAAATAGC
		L-*gadA*-F	AATGTGCAACTTGTCATGGT
		L-*gadA*-R	CGTTATGTTACATTTCGAACTCCTTAAATT
		R-*gadA*-F	GTTCGAAATGTAACATAACGTTGTAAAAAC
		R-*gadA*-R	TCCCTTGACACGAATACAAA
		T-*gadA*-F	ACTGATGCCATTGCTGAATT
		T-*gadA*-R	TCTCTACTACAGTGATGAAC
	*proB*	pTarget-*proB*	ATACTAGTCGAGTTTTACCACCAGCGTCGTTTTAGAGCTAGAAATAGC
		L-*proB*-F	CAGTATCACTCTCTGCTTTA
		L-*proB*-R	CTGCTCCTTACATGATTCTCTGCCATTCAA
		R-*proB*-F	GAGAATCATGTAAGGAGCAGGCTGATGCTG
		R-*proB*-R	TTAGTGCGACACGTTTCTTT
		T-*proB*-F	AGGTTACGACCATAATCGAA
		T-*proB*-R	TTATCCATACGCAGCATTTC
*In situ* promoter replacement	*rhtA*	pTarget-*rhtA*	TAATACTAGTGCGCATTTTAGTCAAAACGGGTTTTAGAGCTAGAAATAGC
		L-*rhtA*-F	TGTCGTGTTTGTAGGTCGGGTATTC
		L-*rhtA*-R	ACCACACATTATACGAGCCGGATGATTAATTGTCAACGTTCTGTTACATGAAATGG
		R-*rhtA*-F	CGGCTCGTATAATGTGTGGTCACAAAGGAGATATACATGCCTGGTTCATTACGTAA
		R-*rhtA*-R	ACTTAAAATGTAAATAGCCCAACAA
		T-*rhtA*-F	AAAGTGATTAGAAGCGGTAA
	*eamA*	pTarget-*eamA*	TAATACTAGTCAAAAGATGAAATTCAGAGGGTTTTAGAGCTAGAAATAGC
		L-*eamA*-F	TGCGCTTTACCCAAATTTGA
		L-*eamA*-R	ACCACACATTATACGAGCCGGATGATTAATTGTCAATTTTTGCCATGCTATTTCTT
		R-*eamA*-F	CGGCTCGTATAATGTGTGGTCACAAAGGAGATATAC ATGTCGCGAAAAGATGGGGT
		R-*eamA*-R	ACCAGACTGTGAATCATGGT
		T-*eamA*-F	TCACCATCACATATTGTGAA
	*aspA*	pTarget-*aspA*	TAATACTAGTTCGATGCAGGGGATAATCGTGTTTTAGAGCTAGAAATAGC
		L-*aspA*-F	TCATCATCCTTTCGCTGGTACTCAC
		L-*aspA*-R	ACCACACATTATACGAGCCGGATGATTAATTGTCAATCTGGATCACTTTAAGTGTC
		R-*aspA*-F	CGGCTCGTATAATGTGTGGTCACAAAGGAGATATACATGTCAAACAACATTCGTAT
		R-*aspA*-R	CTTCTTCTTTCAGCAGGATGCTGAA
		T-*aspA*-F	TGTTATGCGTTCTGTTACTG
	*glk*	pTargetF-*glk*	TAATACTAGTAAGAATTATTTTGACTTTAGGTTTTAGAGCTAGAAATAGC
		L-*glk*-F	CAGCAAGACCGAGAATTAAT
		L-*glk*-R	CATTATACGAGCCGGATGATTAATTGTCAAATACCTGGGGGCATAACAAC
		R-*glk*-F	GTATAATGTGTGGTCACAAAGGAGATATACATGACAAAGTATGCATTAGT
		R-*glk*-R	ATAATGGCCTCTTCTTCACT
		T-*glk*-F	TTTGCGAAAATATCAACGCC
	*gltB*	pTarget-*gltB*	TAATACTAGTCCGATAAGTTGGAAATCCGCGTTTTAGAGCTAGAAATAGC
		L-*gltB*-F	TTTTGCCCATAACGACGGGT
		L-*gltB*-R	CATTATACGAGCCGGATGATTAATTGTCAAATGGCAAGCTTATTGGTACA
		R-*gltB*-F	GTATAATGTGTGGTCACAAAGGAGATATAC ATGACACGCAAACCCCGTCG
		R-*gltB*-R	TTTGCTCAATGCGTGGCAGA
		T-*gltB*-F	AAACGAGGCAACATTACAGA
		TrcV^*b*^	GTGACCACACATTATACGAGCCGGATGA

a Each pTarget primer shares the same reverse primer, pTarget R-common, listed in Table 4.

b TrcV represents a primer complementary to the *trc* promoter.

### Culture conditions.

MS medium was used for l-homoserine production by E. coli W3110-derived strains, consisting of (per liter) 40 g glucose, 17 g (NH_4_)_2_SO_4_, 4 g yeast extract, 1 g KH_2_PO_4_, 1 g MgSO_4_, 25 g CaCO_3_, 5 mg FeSO_4_·7H_2_O, 5 mg MnSO_4_·7H_2_O, and 5 mg ZnSO_4_. Shake flask fermentation was performed in 500-ml flasks containing 20 ml MS medium at 28°C for 48 h at 150 rpm. When necessary, the medium was supplemented with 50 μg/ml chloramphenicol or kanamycin. l-Methionine and l-threonine were supplemented to final concentrations of 0.05 and 0.2 g/liter. Additionally, l-lysine was added to MS medium at final concentrations ranging from 0 to 0.5 g/liter (0, 0.0125, 0.025, 0.05, 0.125, 0.25, and 0.5 g/liter), depending on the experiment, as described in the text. For expression of the CRISPRi system, strains were induced with 0.1 mM IPTG (isopropyl-β-d-thiogalactopyranoside) when the OD_600_ reached 0.4 to 0.6. The cells were cultivated aerobically at 28°C in a shaking incubator at 150 rpm and supplemented with 50 μg/ml antibiotics when necessary.

In fed-batch fermentation, a 5-liter bioreactor (Baoxing, Shanghai, China) was used for l-homoserine, with an effective working volume of 2.0 liters. The medium for fed-batch fermentations consisted of (per liter) 15 g glucose, 14 g (NH_4_)_2_SO_4_, 2 g yeast extract, 2 g KH_2_PO_4_, 0.5 g MgSO_4_, 0.5 g l-threonine, 0.2 g l-methionine, 0.1 g l-lysine, 5 mg FeSO_4_·7H_2_O, 5 mg MnSO_4_·7H_2_O, and 5 mg ZnSO_4_. The feeding medium contained 500 g/liter glucose, 12.5 g/liter KH_2_PO_4_, 10.0 g/liter NaHCO_3_, 4 g/liter l-threonine, 1 g/liter l-methionine, and 0.5 g/liter l-lysine, and the feed was started when the residual glucose concentration was below 10 g/liter. The fermentation temperature was maintained at 28°C, and the pH was adjusted to 6.8 with 40% ammonia water throughout the process. The dissolved oxygen level was maintained above 20% by adjusting aeration and agitation rates.

### Analytical method.

The OD_600_ was measured using an Eppendorf BioPhotometer D30 (Amersham Biosciences, Uppsala, Sweden). Concentrations of glucose were determined by using a glucose analyzer (YSI model 2300; Xylem Inc., Rye Brook, NY, USA) with an IC Sep Ion-300 column (Transgenomic, San Jose, CA, USA). A Sykam S-433D amino acid analyzer (Sykam, Munich, Germany) was used to determine the concentrations of amino acids. By-product measurement was performed with an Aminex HPX-87H column (300 by 7.8 mm), using 5 mM H_2_SO_4_ as the mobile phase, with a flow rate of 0.6 ml/min and detection via refractive index or UV absorption at 210 nm (6).

### RT-qPCR for mRNA quantification.

E. coli strains W3110 and HS33 were cultured for 18 h in 20 ml MS medium at 150 rpm and 28°C. Samples were collected during the exponential growth phase, prepared by centrifugation, and flash frozen in liquid nitrogen and were sent to Tsingke Biological Technology (Wuhan, China) for RT-qPCR analysis with 16S rRNA as a control for normalization between samples. The fold changes of target genes were calculated as 2^−ΔΔ^*^CT^* according to the method of Schmittgen and Livak (52).

### Metabolome analysis.

Metabolomics was applied to characterize the biological variation of the intercellular metabolites between E. coli W3110 and HS33. The samples were collected from cultures grown in 6 technical replicates per strain during the exponential growth phase. The cells were washed twice with ice-cold phosphate-buffered saline (PBS) and flash frozen in liquid nitrogen for further metabolite extraction. Statistical analysis of metabolite profiles was performed by BGI Tech Solutions Co., Ltd. (Shenzhen, China). The intracellular metabolite concentrations of E. coli W3110 were used as a control for normalization between samples. The detailed protocol of the liquid chromatography-mass spectrometry (LC-MS) system is described in the supplemental material. The screened metabolomics data were subjected to partial least squares discrimination analysis (PLS-DA). Significantly changed metabolites (SCM) (defined as follows: base VIP [variable important for the projection], >1.0; fold change, >1.20 or <0.83; *q*-value,  <0.05) were selected for subsequent chemical structure identification.

### Statistical analysis.

All experiments were conducted in triplicate. The data were averaged and are presented as means ± standard deviations (SD). *P* values of <0.01 were considered statistically significant. All the figures were prepared using Origin Software version 8.0 (OriginLab Corp., Northampton, MA, USA).

### Data availability.

The raw data supporting the conclusions of this article will be made available, without undue reservation, to any qualified researcher.

## Supplementary Material

Supplemental file 1
