# Supplementary material for: Multiplex Design of the Metabolic Network for Production of l-Homoserine in Escherichia coli
Source: Appl Environ Microbiol. 2020 Oct 1;86(20):e01477-20. doi: 10.1128/AEM.01477-20 (PMC7531971; doi:10.1128/AEM.01477-20)
Supplement: Supplemental file 1 [file AEM.01477-20-s0001.pdf]

Supplemental Material

Multiplex design of metabolic network for production of L-homoserine in *Escherichia coli*

Peng Liu[1,2], Bo Zhang[1,2], Zhen-Hao Yao[1,2], Zhi-Qiang Liu*[,1,2] and Yu-Guo Zheng[1,2]

[1]The National and Local Joint Engineering Research Center for Biomanufacturing of Chiral Chemicals, Zhejiang University of Technology, Hangzhou 310014, P. R. China

[2]Key Laboratory of Bioorganic Synthesis of Zhejiang Province, College of Biotechnology and Bioengineering, Zhejiang University of Technology, Hangzhou 310014, China

* Corresponding author:

Tel: +86-571-88320614, Fax: +86-571-88320630, E-mail: microliu@zjut.edu.cn

Supplemental Methods

Genetic manipulation of *E. coli* derivatives using CRISPR-Cas9

Firstly, donor DNA with 500-bp homologous arms at upstream and downstream of the target gene was designed. Then these two homology arms containing the trc promoter or gene to be inserted were fused together by overlap-extension PCR. The PCR products were purified by using the AxyPrep PCR Cleanup Kit (Axygene Biotech Ltd., Hangzhou, China). In terms of transformation, the mixture of donor DNA (dsDNA and ssDNA, 400 ng) and corresponding pTarget (100 ng) were added to 100 μl electrocompetent cells and a Bio-Rad MicroPulser (Bio-Rad, Hercules, CA, USA) was used for electroporation (0.2 cm cuvette, 2.5 kV). Then the strain was regenerated at 30 °C for 3 h with 1 ml LB medium prior to plating. The edited colony was achieved by individual bacterial colonies PCR and sequencing. Positive colonies in which the donor DNA replaced the target gene were cultivated in LB and induced by IPTG (0.5 mM) to express sgRNA to cut the corresponding pTarget using Cas9. The pCas was inactivated by heating the culture to 37 ℃.

Metabolome Analysis

All samples were acquired by the LC-MS system followed machine orders. Firstly, all chromatographic separations were performed using an ultra-performance liquid chromatography (UPLC) system (Waters, UK). An ACQUITY UPLC HSS T3 column (100 mm*2.1 mm, 1.8μm, Waters, UK) was used for the reversed phase separation. The column oven was maintained at 50 °C. The flow rate was 0.4 ml/min and the mobile phase consisted of solvent A (water + 0.1% formic acid) and solvent B (methanol + 0.1% formic acid). Gradient elution conditions were set as follows: 0 - 2 min，100% phase A; 2 - 11 min，0% to 100% B; 11 - 13 min，100% B; 13 - 15 min，0% to 100% A. The injection volume for each sample was 5 μL.

A high-resolution tandem mass spectrometer Xevo G2 XS QTOF (Waters, Manchester, UK). was used to detect metabolites eluted form the column. The Q-TOF was operated in both positive and negative ion modes. For positive ion mode, the capillary and sampling cone voltages were set at 3.0 kV and 40.0 V, respectively. For negative ion mode, the capillary and sampling cone voltages were set at 2.0 kV and 40.0 V, respectively. The mass spectrometry data were acquired in Centroid MSE mode. The TOF mass range was from 50 to 1200 Da and the scan time was 0.2 s. For the MS/MS detection, all precursors were fragmented using 20 - 40 eV, and the scan time was 0.2 s. During the acquisition, the LE signal was acquired every 3 s to calibrate the mass accuracy. Furthermore, in order to evaluate the stability of the LC-MS during the whole acquisition, a quality control sample (Pool of all samples) was acquired after every 10 samples.

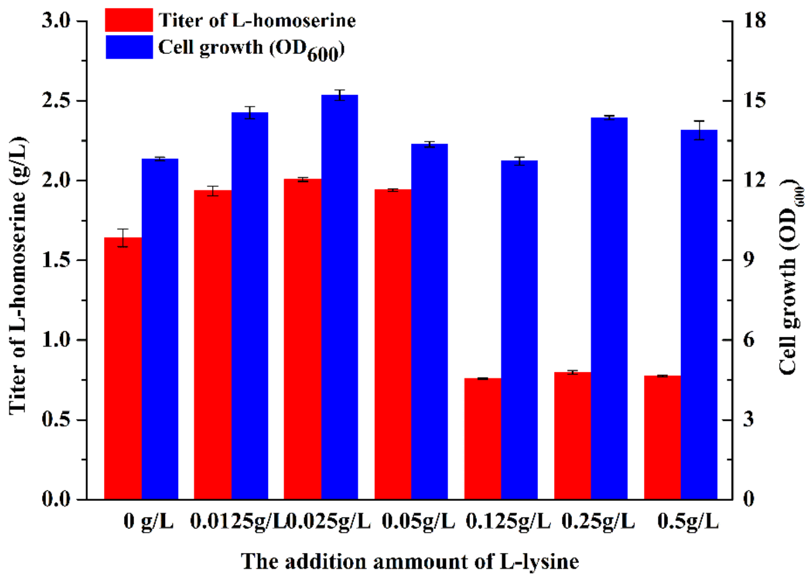

Figure S1. The influence of L-lysine addition on L-homoserine production and growth.

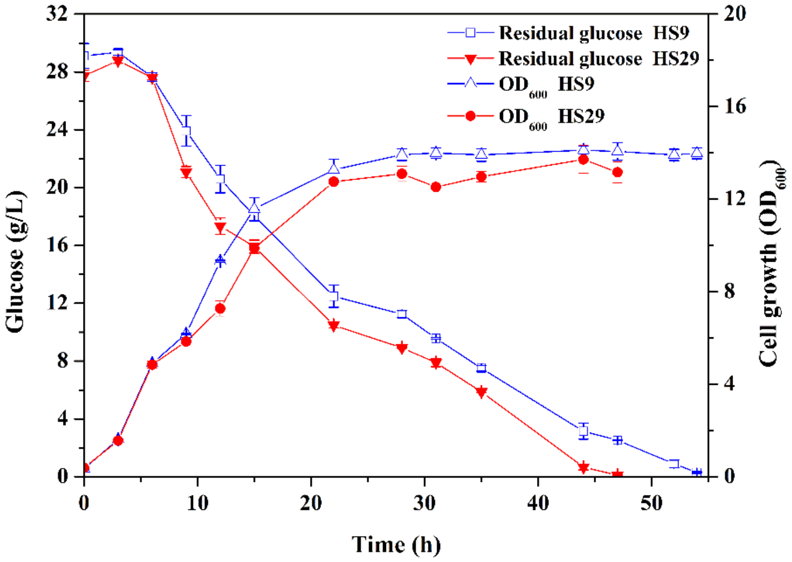

Figure S2. Batch fermentation profiles of HS9 and HS29.

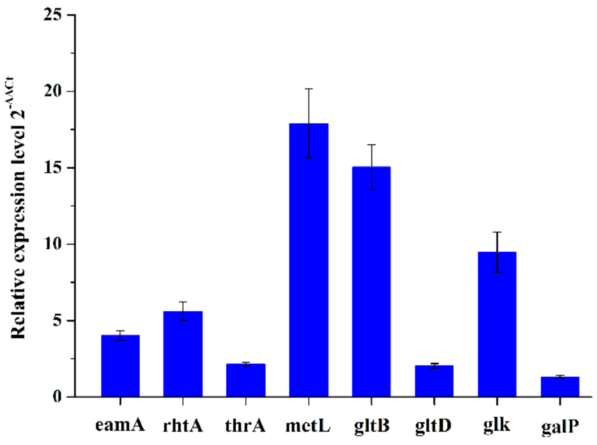

Figure S3. Transcriptional levels of genes involving L-homoserine biosynthesis characterized by RT-qPCR.
